# Therapeutic management of severe spinal cord decompression sickness in a hyperbaric center

**DOI:** 10.3389/fmed.2023.1172646

**Published:** 2023-09-08

**Authors:** Benjamin Simonnet, Romain Roffi, Henri Lehot, Jean Morin, Arnaud Druelle, Lucille Daubresse, Pierre Louge, Sébastien de Maistre, Emmanuel Gempp, Nicolas Vallee, Jean-Eric Blatteau

**Affiliations:** ^1^Department of Diving and Hyperbaric Medicine, Sainte-Anne Military Hospital, Toulon, France; ^2^Military Institute of Biomedical Research (IRBA), Subaquatic Operational Research Team (ERRSO), Toulon, France

**Keywords:** diving, decompression sickness, bubbles, spinal cord, neurological sequelae, hyperbaric oxygen therapy, helium, lidocaine

## Abstract

**Introduction:**

Spinal cord decompression sickness (scDCS) unfortunately has a high rate of long-term sequelae. The purpose of this study was to determine the best therapeutic management in a hyperbaric center and, in particular, the influence of hyperbaric treatment performed according to tables at 4 atm (Comex 30) or 2.8 atm abs (USNT5 or T6 equivalent).

**Methods:**

This was a retrospective study that included scDCS with objective sensory or motor deficit affecting the limbs and/or sphincter impairment seen at a single hyperbaric center from 2010 to 2020. Information on dive, time to recompression, and in-hospital management (hyperbaric and medical treatments such as lidocaine) were analyzed as predictor variables, as well as initial clinical severity and clinical deterioration in the first 24 h after initial recompression. The primary endpoint was the presence or absence of sequelae at discharge as assessed by the modified Japanese Orthopaedic Association score.

**Results:**

102 divers (52 ± 16 years, 20 female) were included. In multivariate analysis, high initial clinical severity, deterioration in the first 24 h, and recompression tables at 4 atm versus 2.8 atm abs for both initial and additional recompression were associated with incomplete neurological recovery. Analysis of covariance comparing the effect of initial tables at 2.8 versus 4 atm abs as a function of initial clinical severity showed a significantly lower level of sequelae with tables at 2.8 atm. In studying correlations between exposure times to maximum or cumulative O2 dose and the degree of sequelae, the optimal initial treatment appears to be a balance between administration of a high partial pressure of O2 (2.8 atm) and a limited exposure duration that does not result in pulmonary oxygen toxicity. Further analysis suggests that additional tables in the first 24–48 h at 2.8 atm abs with a Heliox mixture may be beneficial, while the use of lidocaine does not appear to be relevant.

**Conclusion:**

Our study shows that the risk of sequelae is related not only to initial severity but also to clinical deterioration in the first 24 h, suggesting the activation of biological cascades that can be mitigated by well-adapted initial and complementary hyperbaric treatment.

## Introduction

Among the serious pathologies specific to SCUBA diving, that require hospitalization, decompression sickness (DCS) is the most frequent and presents a significant morbidity with sometimes poor neurological prognosis (1–3). DCS encountered by divers is caused by the formation of bubbles from inert gas initially dissolved in the tissue during hyperbaric exposure. DCS injuries are rare with a prevalence of 1 to 3 per 10,000 dives (3). However, they often occur despite no violation of decompression procedures which limit their prevention. Spinal cord DCS, which presents with neurological symptoms, is the most frequent, accounting for about 50% of clinical DCS forms, and about 30% of long-term neurological sequelae (4, 5). The pathophysiological mechanisms are not fully understood, but result in localized spinal cord ischemia initially triggered by the formation of tissue or vascular bubbles (6).

The hospital management recommended by the most recent consensus conference on hyperbaric medicine is based on an initial compression of 2.8 or 4 absolute atmospheres (atm abs), without being able to choose between these two tables in the absence of sufficient scientific data (7).

**Figure 1:**
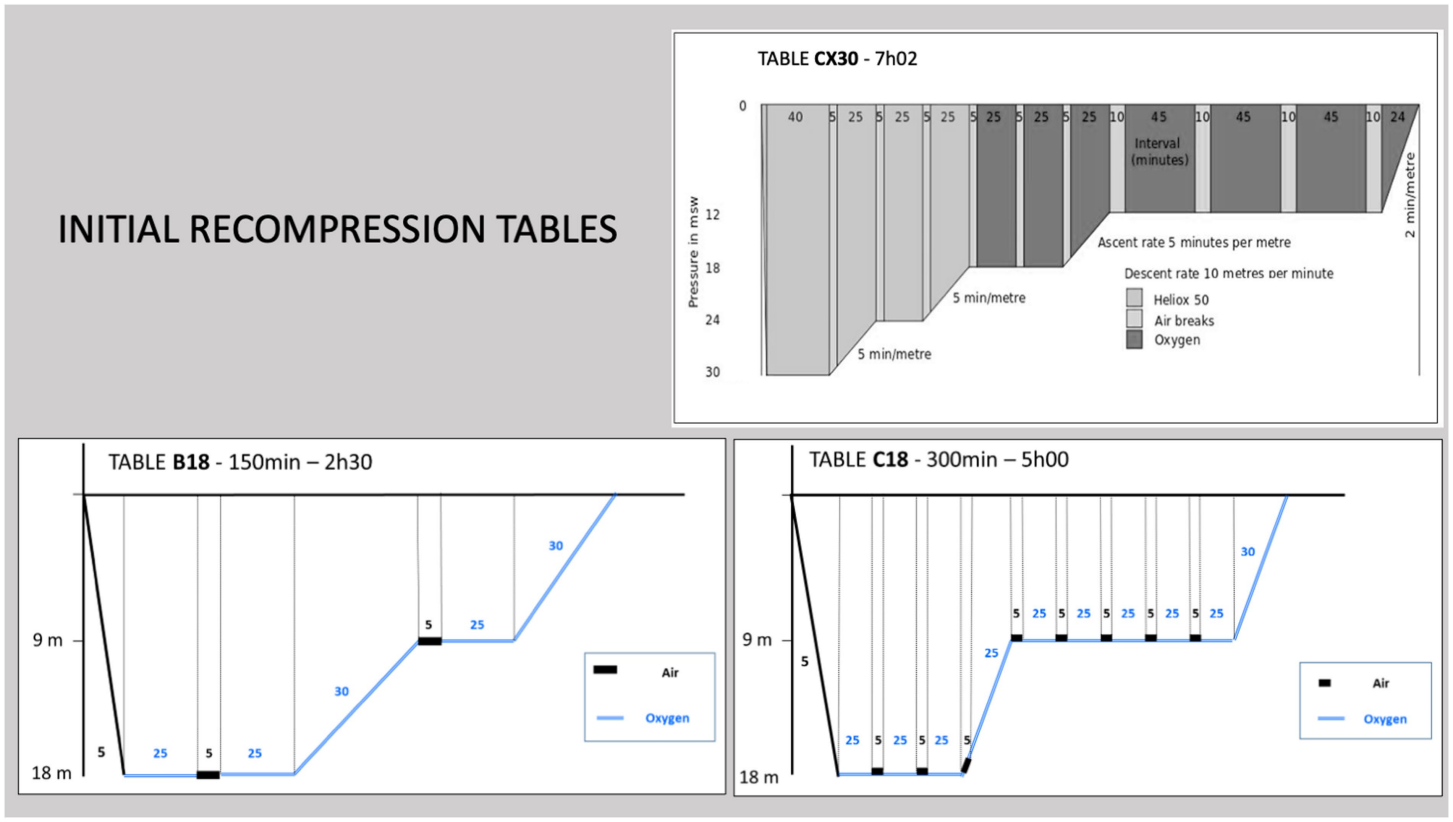
Initial recompression tables. When the injured diver had a MEDSUBHYP score of initial severity ≤7, a B18 table was performed, whereas a C18 table or Comex 30 table was used for an initial score > 7.

At the Department of Diving and Hyperbaric Medicine of the Sainte Anne Military Hospital, Toulon, France, the initial compression of spinal cord DCS was previously based on a 4 atm abs table (Figure 1), using 50% Heliox (50% oxygen and 50% helium) and oxygen as the breathing gas. Currently, the preferred oxygen tables are at 2.8 atm abs, i.e., tables equivalent to US Navy tables 5 or 6 (Figure 1). The use of these tables at lower pressure levels allows the use of pure oxygen throughout most of the table.

**Figure 2:**
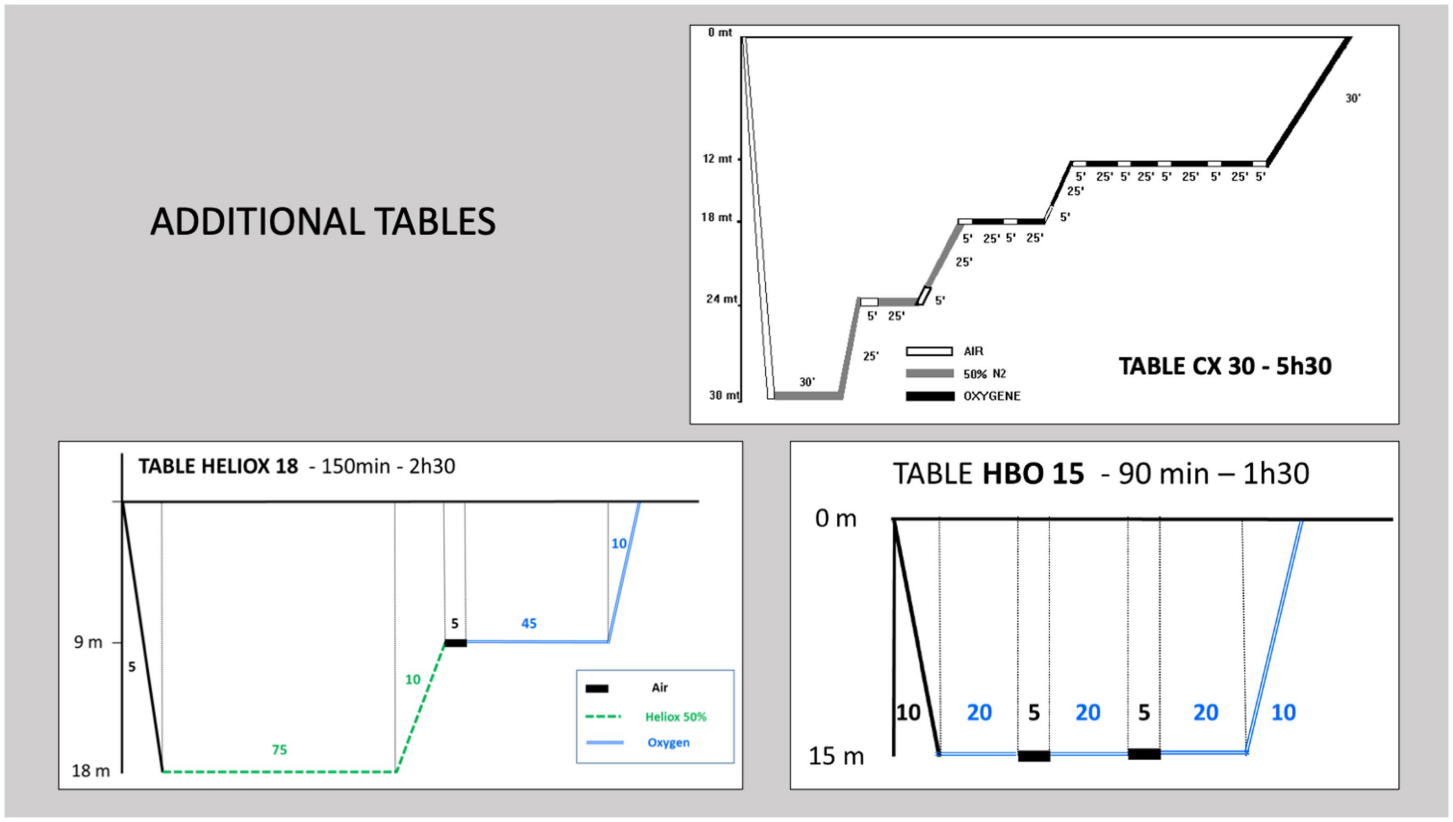
Additional recompression tables. Heliox tables at 18 m (2.8 atm abs) and 30 m (4 atm abs) or 100% O2 tables were performed in the first 24–48 h after the initial table followed by consolidation sessions with daily 100% O2 tables at 15 m (2.5 atm abs).

After this initial recompression, additional tables at 2.5 atm abs are often performed if clinical symptoms persist. In recent years, protocols have evolved with the introduction of additional sessions in the first 48 h for the most severe patients. Additional Heliox tables at 4 atm abs have been used and, more recently, additional Heliox tables at 2.8 atm abs have been performed (Figure 2). The aim of these additional Heliox sessions is to benefit from a possible neuroprotective effect of helium (8), which would improve neurological recovery (9).

For severe symptomatic cases, in addition to hyperbaric treatment, a lidocaine protocol has been implemented for its neuroprotective effects in aeroembolic stroke, although its efficacy remains controversial (10–12). The protocol consists of a continuous infusion at an antiarrhythmic dose with continuous cardiac rhythm monitoring.

Prolonged treatment with fluoxetine for a period of 3–6 months is currently proposed not only for its antidepressant effect, but also to stimulate brain plasticity and promote functional recovery in animal models of DCS (13, 14).

Therefore, it seemed important to us to compare these hyperbaric and medical treatments in order to optimize the treatment of this pathology, which can cause significant disability.

The first objective of this study is to compare the effectiveness of initial recompression tables at 2.8 vs. 4 atm abs on the outcome of spinal cord DCS in a series of injured divers with severity criteria during the first 24 h.

The secondary objective is to evaluate the benefit of medical therapy associated with recompression and the value of supplemental hyperbaric tables ≤2.8 vs. 4 atm abs performed in the first 48 h.

## Methods

### Inclusion criteria

All patients admitted to the hyperbaric center of the Sainte-Anne military hospital, Toulon, France, between 2010 and 2020 were included in the study if they presented with unilateral or bilateral neurological signs affecting the upper and/or lower limbs or with sphincter impairment within the first 24 h. After reviewing all records, we excluded patients with only subjective signs and symptoms suggestive of brain injury, including cognitive signs consistent with a diagnosis of cerebral DCS or cerebral aeroembolism secondary to pulmonary barotrauma.

### Data collection

At the time of patient presentation, the diving medicine specialists at the hyperbaric centre systematically completed a DCS database form with the following information:

- Dive: total dive time, maximum depth, dive level, gas mix (air, nitrox or trimix), equipment (open circuit or rebreather), repetitive dives (dives made in the previous 6 h), procedural errors (rapid ascent, omission of decompression stops).
- Time to recompression (from onset of symptoms to first recompression).
- Clinical data: age, sex, history of DCS and clinical status on admission, assessed by the French Society of Diving and Hyperbaric Medicine score (MEDSUBHYP score), which combines several of these clinical data (Table 1) and allows estimation of the initial severity of neurological DCS (4, 15). Clinical deterioration in the first 24 h after initial recompression was also analysed, independent of clinical status on admission.
- Hyperbaric treatments:
- initial tables at 2.8 atm abs (O2 100%) or 4 atm abs (Heliox 50% and O2 100%);
- additional tables within the first 48 h ≤ 2.8 atm abs (O2 100% 2.5 atm abs or Heliox 50% 2.8 atm abs) or 4 atm abs (Heliox); and
- consolidation tables (O2 100% 2.5 atm abs).

**Table 1:** The MEDSUBHYP score of initial clinical severity and its numerical weighting.

		0	1	2	3	4	5	6
Age > 42	*No*	X						
	*Yes*		X					
Back pain	*No*	X						
	*Yes*		X					
Clinical course before recompression	*Better*	X						
	*Stable*				X			
	*Worse*						X	
Objective sensory deficit	*No*	X						
	*Yes*					X		
Motor impairment	*None*	X						
	*Paresis*					X		
	*Paraplegia*						X	
Bladder dysfunction	*No*	X						
	*Yes*							X

The choice of initial recompression was generally based on initial clinical severity. If the casualty had a MEDSUBHYP score ≤ 7, recompression was generally performed at 2.8 atm abs (O2 100%) for 2 h 30 min (equivalent to US Navy table 5). If the initial score was >7, a procedure at 2.8 atm abs (O2 100%) for 5 h (equivalent to US Navy table 6) or 4 atm abs for 7 h (equivalent to Comex 30 table) was preferred. The ratio of O2 partial pressure equal to 2.8 atm to total table duration is 0.33 for the short table at 2.8 atm, 0.25 for the long table at 2.8 atm and 0.12 for the table at 4 atm abs (Figure 1).

However, over this 10-year period, adherence to this protocol may have varied from practitioner to practitioner, depending on the analysis and habits of each hyperbaric physician.

In the event of deterioration or lack of efficacy of the initial recompression, additional tables were performed within the first 48 h using Heliox tables at 4 atm abs (with one to two sessions), Heliox tables at 2.8 atm abs (two sessions) or oxygen tables (O2 100%, one session/day) at 2.5 atm abs (Figure 2). Supplementary O2 tables for 90 min at 2.5 atm abs (one session/day) were performed on the following days in case of residual symptoms until recovery allowed discharge or clinical stabilisation followed by transfer to a rehabilitation centre. The decision to discontinue or continue sessions was subject to peer review by the physicians at the hyperbaric centre.

- Means of evacuation: helicopter or by road.
- Prehospital treatment: which almost systematically includes normobaric oxygen, hydration (*per os* or by vascular filling) and oral administration of aspirin (250 mg);
- Hospital therapy: vascular filling (infusion of isotonic salt serum, 1 L/5 h), corticosteroids (methylprednisone, intravenous, 1 mg/kg), lidocaine (intravenous infusion, 2 mg/min for 36 h) and fluoxetine (*per os*, 20 mg/d.).

The primary end point was the clinical status of the patient at discharge from the hyperbaric centre, as assessed by the modified Japanese Orthopaedic Association (mJOA) score (16). This score allows a quantification on a total of 17 points. The lower the score, the more severe the deficits; a score = 16 or 17 indicates normal function (Table 2).

**Table 2:** Spinal cord injury assessment score called “modified Japanese Orthopedic Association—mJOA Score” and its numerical weighting.

Criterion	Points
**I. Upper extremity motor function**	
Unable to feed oneself	0
Unable to use knife and fork, able to eat with a spoon	1
Able to use knife and fork with much difficulty	2
Able to use knife and fork with slight difficulty	3
No disability	4
**II. Lower extremity motor function**	
Unable to walk	0
Can walk on flat floor with walking aid	1
Can walk up and/or down stairs with handrail	2
Lack of stability and smooth gait	3
No disability	4
**III. Sensory function**	
**A. Upper extremity**	
Apparent sensory loss	0
Minimal sensory loss	1
Normal function	2
**B. Trunk (same as A)**	
**C. Lower extremity (same as A)**	
**IV. Bladder function**	
Complete retention	0
Severe dysfunction	1
Mild dysfunction	2
Normal function	3
**Total score**	**0–17**

### Statistical analysis

Statistical analyses were performed using GraphPad Prism software (version 2020). Based on the distribution of the data, continuous values were expressed as mean ± SD and/or median ± interquartile range. There was no *a priori* sample calculation; all patients available in the database were included in the study. For clinical relevance and ease of interpretation, we decided to dichotomize the outcome variable as the presence or absence of sequelae at discharge, as assessed by a mJOA score < or ≥ 16.

The variables available in the database were integrated into a multiple correspondence analysis (MCA), supplemented by a selection of relevant variables based on the literature review. MCA was used to reduce dimensions by identifying collinearity and redundancy between variables.

A univariate analysis was performed to identify predictors of outcomes, using the χ2 test for categorical variables with Yates correction or the Fisher test for small numbers. For quantitative variables with a normal distribution according to the Agostino & Pearson test, the *t*-test was used. Quantitative variables that did not follow a normal distribution were transformed into qualitative variables by determining discriminatory thresholds through receiver operating curve (ROC) analysis. Variables with a *p*-value ≤0.20 were retained for multivariate analysis with backward elimination logistic regression to control for potential confounders and to identify independent predictors of outcomes. A *p*-value <0.05 was considered significant, and ORs with 95% CIs were reported.

Additional analyses were performed to compare the effect of initial tables on the level of sequelae as a function of initial severity. A two-way analysis of covariance (ANCOVA) was used. The hypotheses of homogeneity of regression lines and homogeneity of variances were tested. Because the assumption of residual normality was not met, the mJoA score was normalized. The Yeo-Johnson transformation with parameter λ = 3.69 was used, with a Pearson normality statistic of *p* = 2.515.

We also compared mJOA sequelae scores and number of additional HBO sessions performed between tables at 2.8 and 4 atm abs by Kruskal-Wallis test in patients with an initial MEDSUBHYP score > 7 to obtain groups of homogeneous initial clinical severity. In this subgroup of patients with initial severity >7, we also looked for correlations (Spearman), (1) to try to determine whether the ratio of oxygen partial pressure equal to 2.8 atm over total table time could be correlated with the level of sequelae (mJOA score), and (2) whether the total cumulative dose of oxygen delivered (based on the calculation of unit pulmonary toxic dose—UPTD) could also be correlated with the level of sequelae.

In addition, to compare the short and long initial tables at 2.8 atm abs, we performed an analysis in the subgroup with the MEDSUBHYP score interval common to these two initial tables, i.e., >7 and < 19.

Further analyses were performed to evaluate the effect of lidocaine and to compare additional tables at 2.8 (Heliox 50%) vs. 2.5 (100% O2) atm performed in the first 48 h in patients with comparable clinical severity after first recompression by calculating the MEDSUBHYP score at 24 h.

## Results

### Selection of variables

Multiple Correspondence Analysis (MCA) was performed on all variables available in the database. The axes F1 (10.8%) and F2 (8.3%) are the most relevant to explain for the model: the variables “number of additional HBO sessions” and “mJOA score” contribute significantly to the construction of F1 and the “MEDSUBHYP score” to the construction of F2.

Prehospital variables including means of evacuation and prehospital treatment (normobaric oxygen, hydration, and oral aspirin) were not included as variables in this study. In fact, these variables have recently been the subject of specific analysis and have shown no influence on the level of neurological sequelae in our center (15). The same is true for diving variables, such as diving level, gas and apparatus.

The hospital treatment variables (corticosteroids and vascular filling) were not included in the analysis because they contributed little to the model and were otherwise administered to more than 90% of the population.

The variable “number of additional HBO sessions”, which is relevant to the description of the model, is strongly correlated with the level of sequelae (mJOAS). To limit the redundancy of the treatment endpoint, we removed this variable from the analysis, as it is less relevant than the estimation of the level of sequelae according to the mJOAS (15). On the other hand, we included the variable “additional tables in the first 48 h” in the analysis to investigate the specific effect of 2.5, 2.8 or 4 atm abs tables performed in the first 48 h.

Finally, patient characteristics (age, gender, history of DCS), clinical parameters (MEDSUBHYP score), dive parameters (duration, maximum depth, repetitive dives) and the different medical or hyperbaric therapies performed at the hyperbaric center were analyzed as predictor variables and included in the univariate analysis (Table 3).

**Table 3:** Results of univariate and multivariate analyses on a series of 102 neurological DCS.

Variables	Sequelae mJOAS <16	No sequelae mJOAS ≥16 (%)	Univariate analysis *p*-value	OR (95% CI)	Multivariate analysis *p*-value	Adj OR (95% CI)
**Gender**						
Woman Male	6 41	14 (70%) 41 (50%)	*p* = 0.11	0.43 (0.15–1.21)	*p* = 0.35	–
**History of DCS**						
No Yes	40 7	50 (56%) 5 (42%)	*p* = 0.36	–	–	–
**Age**						
≤ 52 y > 52 y	23 24	28 (55%) 27 (53%)	*p* = 0.84	–	–	–
**Depth (meters)**						
(T-test)	43 ± 10	39 ± 10	*p* = 0.10	X	*p* = 1.00	–
**Dive (time)**						
≤ 34 min > 34 min	26 21	27 (51%) 28 (65%)	*p* = 0.53	–	–	–
**Repetitive dives**						
No Yes	37 10	48 (56.5%) 7 (41%)	*p* = 0.25	–	–	–
**Initial MEDSUBHYP severity score**						
(T-test)	11 ± 6	9 ± 3	***p*** **= 0.03**	X	***p* = 0.01**	0.84 (0.73–0.96)
**Clinical worsening in the first 24 h**						
No Yes	16 31	37 (70%) 18 (37%)	***p* < 0.001**	0.25 (0.11–0.58)	***p* = 0.004**	0.15 (0.04–0.51)
**Time to recompression**						
< 194 min ≥ 194 min	26 21	33 (56%) 22 (51%)	*p* = 0.63	–	*p* = 0.64	–
**Initial recompression tables**						
2.8 atm abs 4 atm abs	31 16	48 (61%) 7 (30.5%)	***p* = 0.01**	3.54 (1.37–8.91)	***p* = 0.04**	3.87 (1.07–15.1)
**Additional tables in the first 48 h**						
≤ 2.8 atm abs 4 atm abs	29 18	54 (65%) 1 (5%)	***p* < 0.001**	33.5 (5.06–356)	***p* = 0.01**	19.5 (2.90–402)
**Lidocaine**						
Yes No	25 22	15 (37.5%) 40 (64.5%)	***p* = 0.01**	0.33 (0.14–0.77)	*p* = 0.49	–
**Fluoxetine**						
Yes No	10 37	16 (61.5%) 39 (51%)	*p* = 0.37	–	–	–

The level of sequelae was assessed by the modified Japanese Orthopaedic Association Score (mJOAS), i.e., divers presenting sequelae (mJOAS <16) or divers with complete recovery (mJOAS ≥16). A *p*-value <0.05 (in bold) was considered as significant, ORs and adjusted ORs with 95% CIs were reported.

### General description

**Figure 3:**
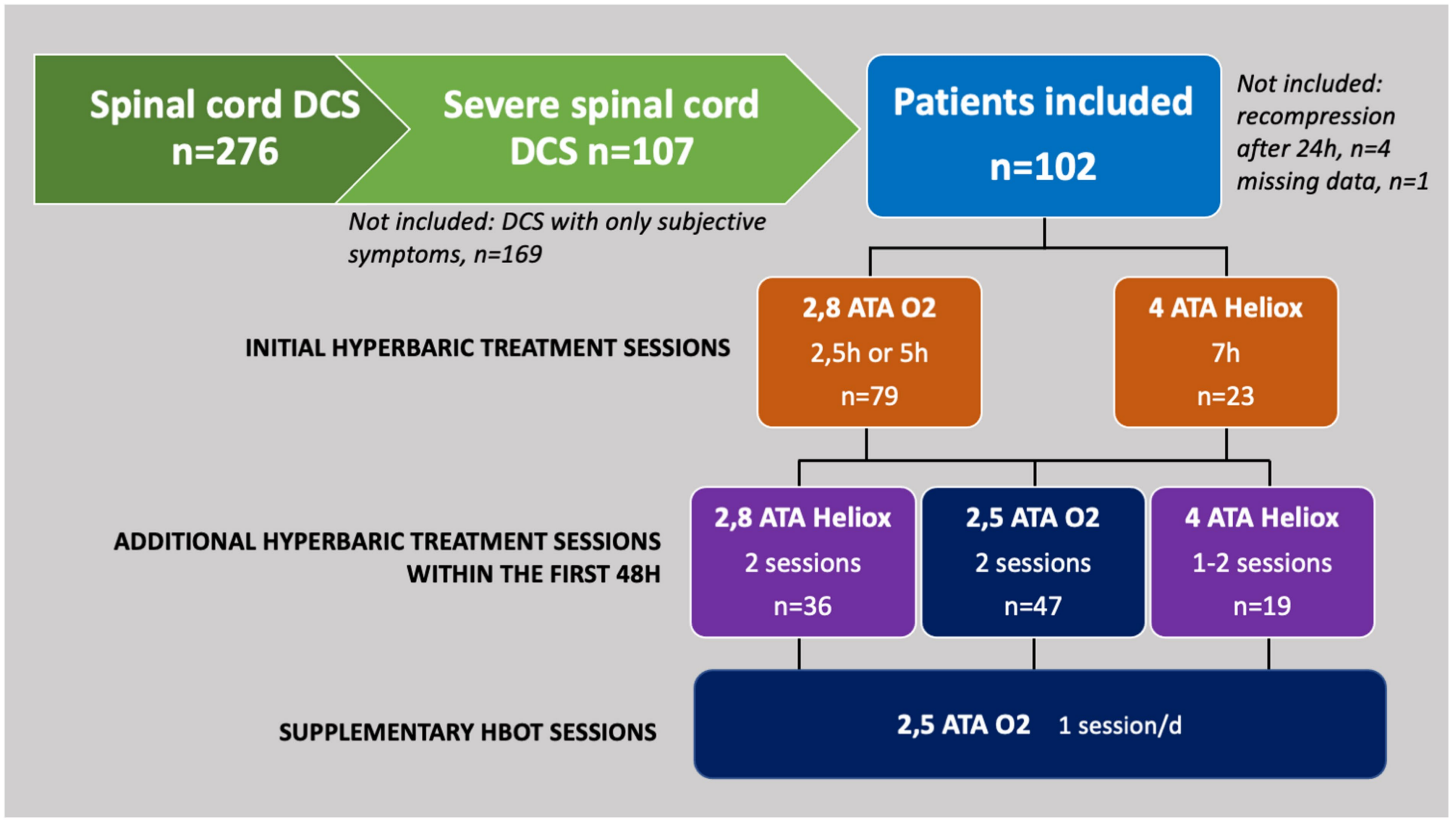
Flow chart describing the inclusion of the 102 subjects and the recompression tables performed.

As described in the flowchart (Figure 3), we included 102 patients, the majority of whom were male, 82 males (80%) and 20 females (20%) with a median age of 52 ± 16 years. 12 patients (12%) had a history of DCS. The median maximum depth was 41 m ± 13 m with a mean dive time of 34 ± 12 min. 17 accidents (17%) occurred during repetitive dives. Procedural errors occurred in only 5 divers (5%) and were not included in the analysis due to their small proportion. The median MEDSUBHYP score was 9 ± 4 and clinical deterioration within 24 h was observed in 49 patients (48%). The median MJOAS at discharge was 16 ± 4 and 47 patients (46%) had neurological sequelae with MJOAS <16. The median time to recompression was 180 ± 147 min with 44 short 2.8 atm abs tables (43%), 35 long 2.8 atm abs tables (34%) and 23 4 atm abs tables (23%). In addition, 98 patients received initial normobaric oxygen therapy (96%), 92 received intravenous hydration (90%), and 102 received aspirin and corticosteroid therapy (100%). After the initial table, 40 patients received a lidocaine protocol (39%) and 36 received a fluoxetine protocol (35%). Finally, 19 patients received additional compression within the first 48 h at 4 atm abs (19%), 36 at 2.8 atm abs (35%), and 47 at 2.5 atm abs (46%).

### Univariate and multivariate analysis

Table 3 shows the results of univariate and multivariate analysis.

Initial severity as assessed by the MEDSUBHYP score and clinical deterioration within the first 24 h were associated with the occurrence of sequelae.

In contrast, initial recompression to 2.8 atm abs versus 4 atm tables was significantly associated with a better neurological prognosis at discharge. We found a similar result for the additional ≤2.8 atm abs vs. 4 atm tables.

In addition, treatment with lidocaine was associated with a worse neurological prognosis, whereas there was no significant difference between the groups with or without fluoxetine.

The following variables with a *p*-value ≤0.20 were included in the multivariate analysis: sex, depth, MEDSUBHYP score, clinical deterioration in the first 24 h, initial tables, additional tables, and lidocaine. The variable “time to recompression” was also included as a “forced” variable because of its significant influence found previously (15, 17). After adjustment, a significant association with the occurrence of sequelae was confirmed for initial severity (MEDSUBHYP score) [OR 0.84 (0.73–0.96); *p* = 0.01] and for clinical worsening in the first 24 h [0.15 (0.04–0.51); *p* = 0.004].

Initial recompression at 2.8 atm abs [OR 3.87 (1.07–15.1); *p* = 0.04] and additional compressions ≤2.8 atm abs [OR 19.5 (2.90–402); *p* = 0.01] were significantly associated with improved neurological prognosis.

### Additional analysis

#### Effect of initial tables on the level of sequelae as a function of initial severity

**Figure 4:**
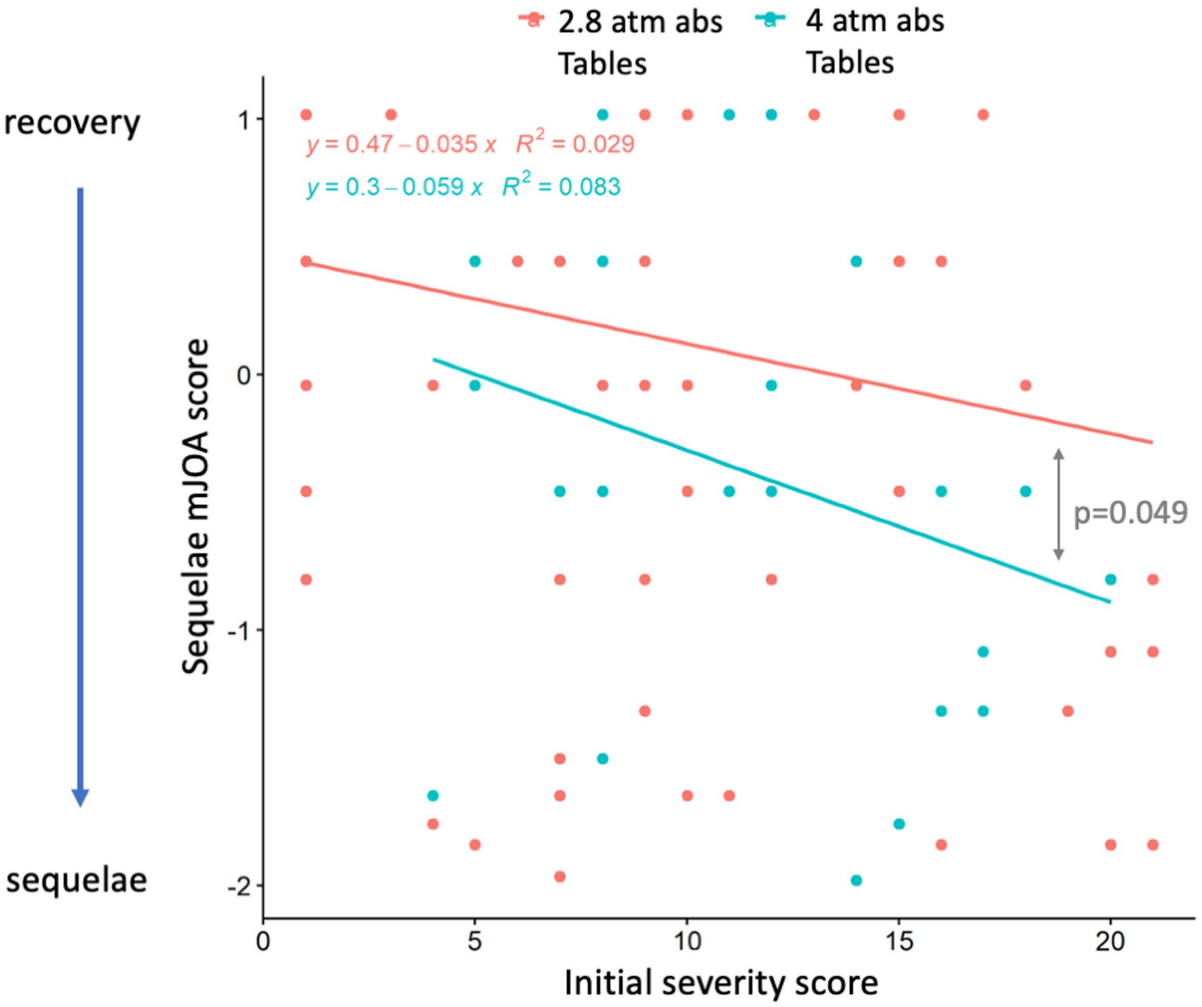
Scatter plot of the 102 DCS divers treated with initial tables at 2.8 atm abs (blue) or 4 atm abs (red) as a function of initial clinical severity (MEDSUBHYP score) and level of sequelae (mJOA score). The mJOA score has been normalized according to the MEDSUBHYP score and according to the two treatments (2.8 and 4 atm). Each point represents one or more divers. The analysis of covariance comparing the regression lines according to the two treatments shows a significant difference between the treatment at 2.8 atm and the treatment at 4 atm abs.

Figure 4 shows DCS cases (*n* = 102) treated with initial tables at 2.8 or 4 atm abs according to initial clinical severity (MEDSUBHYP score) and level of sequelae (mJOA score).

Analysis of covariance comparing the regression lines according to the two treatments showed that there was a significant difference (*p* = 0.049) between the 2.8 and 4.0 atm treatments.

#### Comparison of mJOA sequelae scores between 2.8 and 4 atm abs initial tables

**Figure 5:**
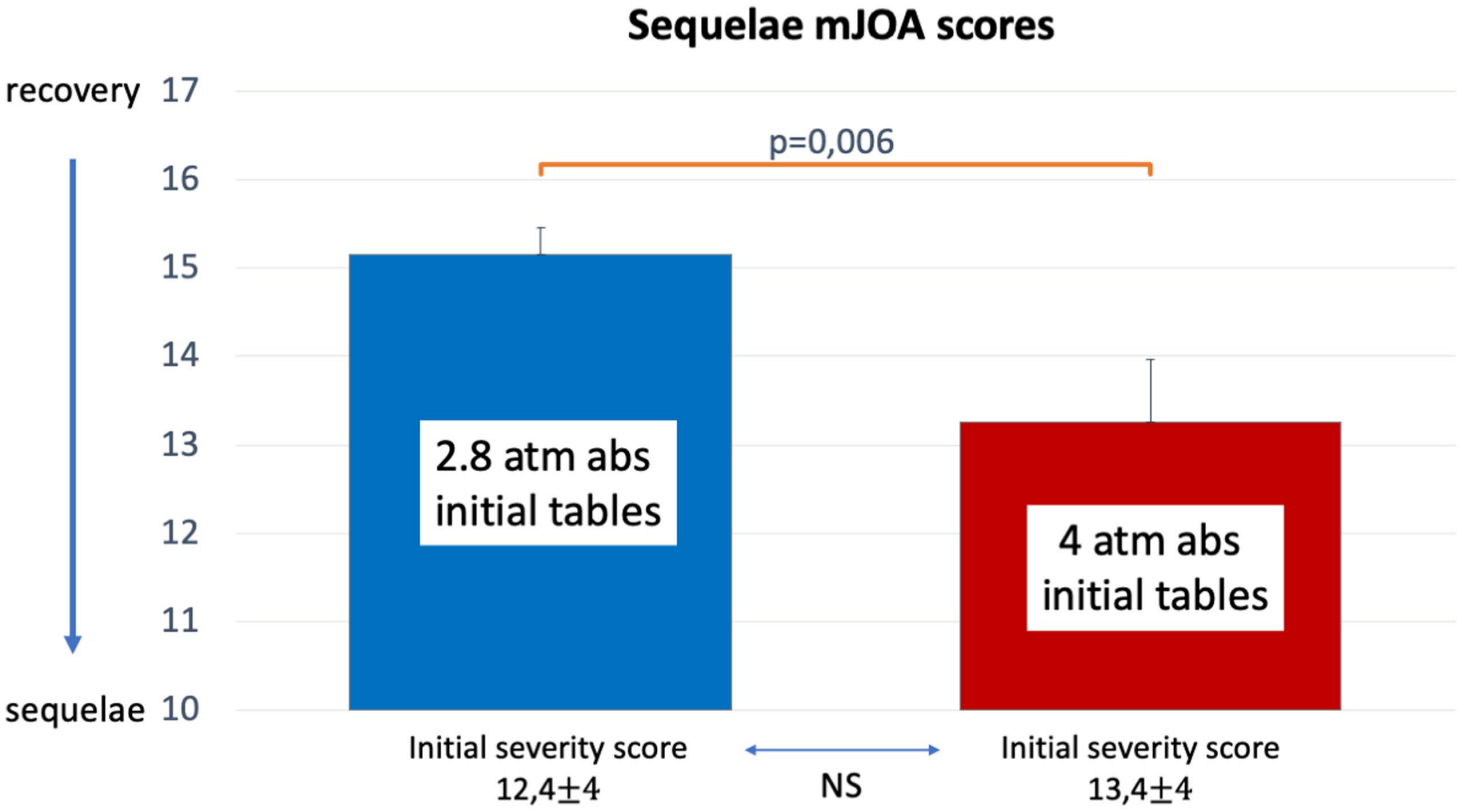
Histograms (mean, standard error and *p*-value) showing mJOA score comparisons between initial tables at 2.8 atm abs or 4 atm abs for DCS injuries with an initial MEDSUBHYP score > 7. The mean values with standard deviation of the MEDSUBHYP scores are specified, with no statistical difference between the two groups.

To compare the effect of initial tables in patients with similar initial severity criteria, we selected patients with a MEDSUBHYP score > 7. In this subgroup, MEDSUBHYP scores did not differ with mean values of 12 ± 4 for 2.8 atm abs tables vs. 13 ± 4 for 4 atm abs tables (*p* = 0.084). There were 37% of patients who had sequelae with the 2.8 atm abs tables (*n* = 60) vs. 70% with the 4 atm abs tables (*n* = 20, *p* = 0.018). The mean mJOA scores were significantly different, with a score of 15 ± 3 for the 2.8 atm abs tables indicating a lower level of sequelae than the score of 13 ± 3.5 for the 4 atm abs tables (*p* = 0.006) (Figure 5). In addition, the mean number of additional HBOT sessions required was 6 ± 4 in the group with initial 2.8 atm abs tables versus 9 ± 3 in those with initial 4 atm abs tables (*p* = 0.003).

In this subgroup of patients with an initial severity >7, we found a significant correlation between the ratio of O2 partial pressure equal to 2.8 atm over total table time and the mJOA score, with a correlation coefficient of 0.19 (*p* < 0.0001), indicating a lower level of sequelae when the ratio is higher. However, we found a significant correlation between the cumulative oxygen dose (UPTD) delivered by the initial table and the mJOA score, with a correlation coefficient of −0.48 (*p* < 0.0001), indicating a lower level of sequelae when the oxygen dose is lower.

#### Comparison of mJOA sequelae scores between short and long initial 2.8 atm abs tables

**Figure 6:**
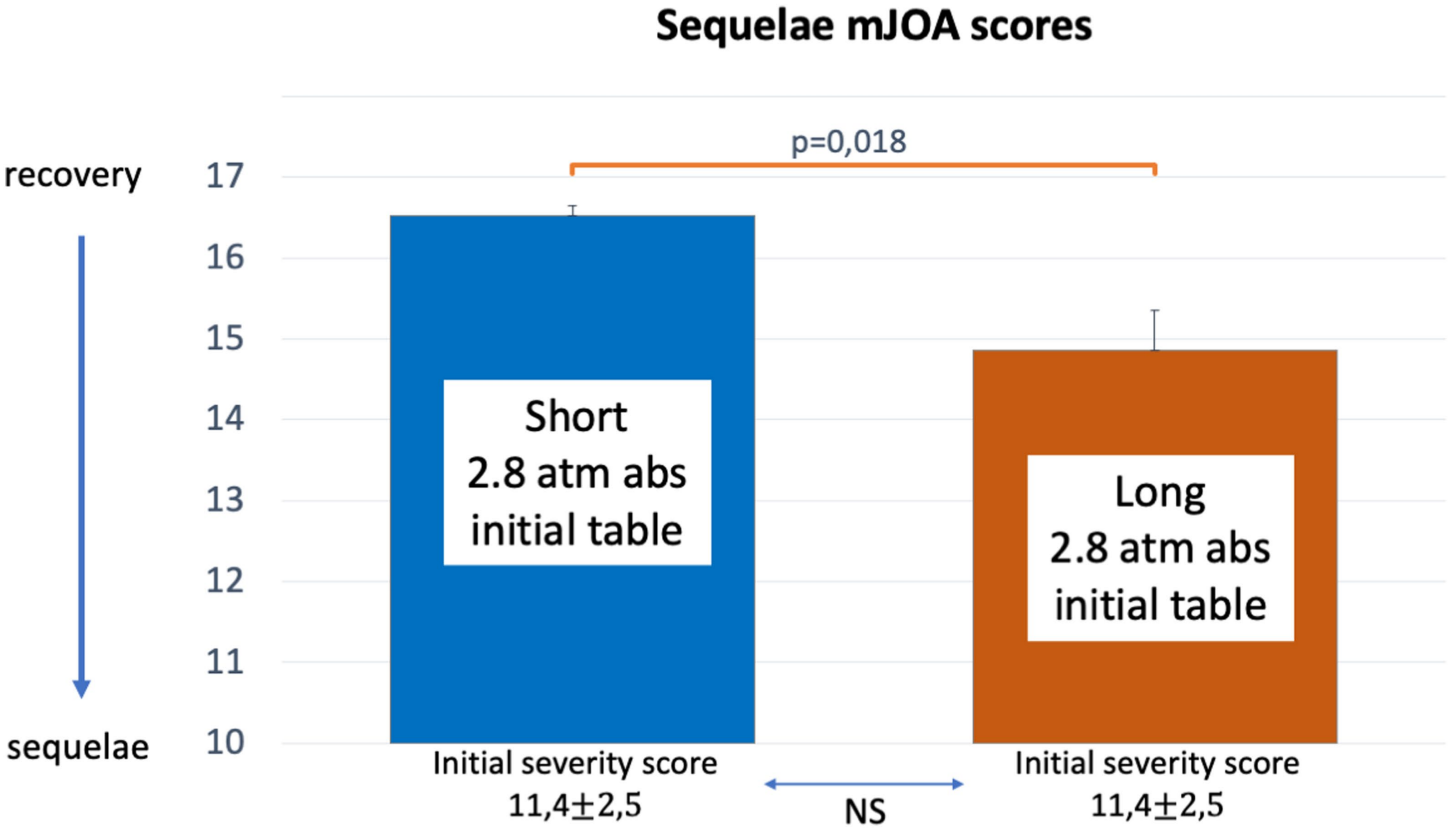
Histograms (mean, standard error and *p*-value) showing mJOA score comparisons between Short and Long initial oxygen tables at 2.8 atm abs for DCS injuries with an initial MEDSUBHYP score > 7 and < 19. The mean values with standard deviation of the MEDSUBHYP scores are specified, with no statistical difference between the two groups.

We compared the efficacy of the long vs. short 2.8 atm abs initial tables on the occurrence of sequelae in patients with the same initial MEDSUBHYP score in the range > 7 and < 19. In this subgroup, MEDSUBHYP scores did not differ with mean values of 11.4 ± 2.5 for both short and long 2.8 atm tables (*p* = 0.99). There were 15% of patients who had sequelae with the short tables (*n* = 27) vs. 41% with the long tables (*n* = 27, *p* = 0.066).The mean mJOA scores were significantly different, with values of 16.5 ± 1 for the short tables indicating a lower level of sequelae than the score of 15 ± 3 for the long tables (*p* = 0.018) (Figure 6).

#### Subgroup analyses after the first therapeutic table

To allow subgroup analyses after the first therapeutic table, we defined clinical severity after the first hyperbaric treatment by calculating the MEDSUBHYP score 24 h after the start of treatment. This score was associated with an adverse neurological prognosis (*p* < 0.0001) with a severity threshold of ≥13 at 24 h.

#### Comparison of additional tables performed in the first 48 h at 2.8 or 2.5 atm abs

**Figure 7:**
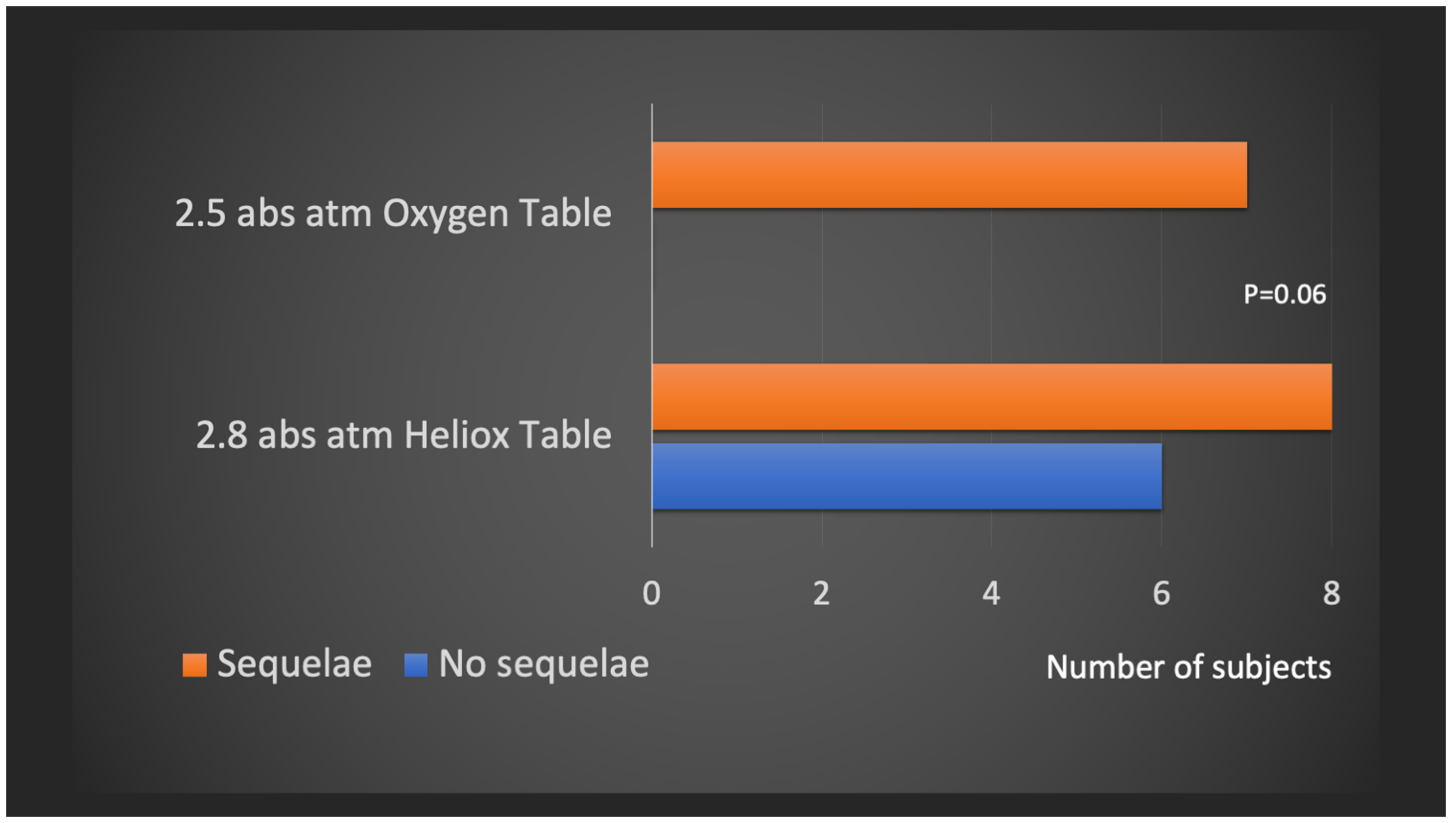
Subgroup analysis of patients with equivalent clinical severity at the end of the first recompression. Comparison of the number of sequellar patients (mJOAS <16) according to the additional hyperbaric treatment performed in the first 24–48 h, i.e., the O2 100% table at 2.5 atm abs vs. the Heliox 50% table at 2.8 atm abs.

This severity score allowed us to compare the value of additional tables in the first 48 h in the subgroup of patients with 24-h severity criteria. Only 57% of patients had sequelae with the Heliox 2.8 atm abs tables versus 100% with the Oxygen 2.5 atm abs tables, a result close to statistical significance (*p* = 0.06) (Figure 7).

#### Effect of lidocaine

**Figure 8:**
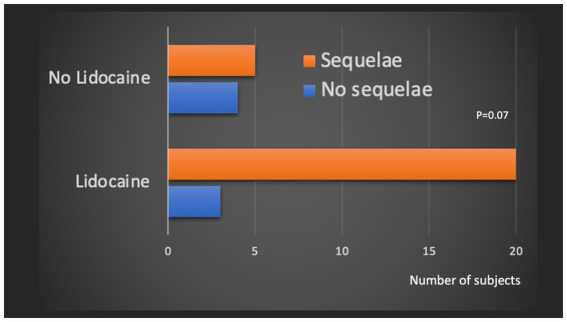
Subgroup analysis of patients with equivalent clinical severity at the end of the first table. Comparison of the number of sequelae patients (mJOAS <16) according to the administration or not of a lidocaine infusion.

We also examined the effect of lidocaine treatment in the subgroup of patients who were severe at 24 h. Lidocaine was administered to 72% of patients with clinical severity criteria according to the MEDSUBHYP score at 24 h. 87% of patients treated with lidocaine had sequelae compared to 55% of patients not treated, but this result was not statistically significant (*p* = 0.07) (Figure 8).

## Discussion

### Risk factors for neurological sequelae

We found that 46% of divers had neurological sequelae with MJOAS <16 at discharge from the hyperbaric center. Usually, the majority of published series describe a lower rate of sequelae in the order of 20%–30% depending on the study for neurological DCS (4, 15, 18).

This finding may be explained by the fact that our study enrolled patients who presented only with initial severity criteria in the first 24 h. Patients with minor symptoms such as paresthesias during this time window were not included.

Furthermore, despite these inclusion criteria, we found that clinical assessment during initial hospital management using the MEDSUBHYP score remained relevant as a prognostic factor for neurological sequelae. The originality of our study is to highlight the importance of clinical evolution at 24 h for neurological prognosis at discharge, independent of initial management and time to recompression. After adjustment, this criterion remained significant. This result confirms for the first time the clinical impression of practitioners in hyperbaric centers that a proportion of patients with spinal cord DCS deteriorate after the first recompression. This suggests that there is an activation of biological cascades that are not stopped despite the elimination or reduction of bullae by HBOT. This paradoxical evolution is probably related to the activation of numerous neurotoxic and inflammatory responses triggered by the initial bullous aggression demonstrated in animal models of DCS (19, 20).

### Initial recompression

#### 2.8 vs. 4 atm abs tables

For a long time, the symptoms of decompression sickness were thought to be a direct result of the formation of nitrogen bubbles in the body and were assumed to be proportional to depth. Historically, recompression was sought at a depth that would relieve the joint pain associated with decompression sickness. The first therapeutic air recompression tables were constructed between 50% and 100% of maximum pressure before slowly ascending to the surface (21). However, as early as 1878, Paul Bert observed from animal experiments that recompression alone did not eliminate the neurological symptoms of decompression sickness (22). The value of using 100% oxygen tables was applied by Benhke in the early 20th century (23). However, it was not until the end of World War II that the first US Navy oxygen tables were used on a large scale with convincing results. These tables of 100% O2 at 2.8 ATA subsequently became the reference method in the US Navy and many other centers (21). The idea of physically neutralizing the bubbles by applying pressures higher than 2.8 atm abs remains a recurring one, but it is then necessary to dilute the oxygen to limit the risk of hyperoxic crisis. Experimental work on carotid gas embolism in anesthetized dogs suggests that bubble disappearance may be favored by pressures greater than or equal to 4 atm abs (24). However, these results have not been subsequently confirmed. In fact, the work of Leitch et al. showed no benefit in favor of pressures above 2.8 atm abs at the same oxygen partial pressure in animal models of gas embolism or spinal cord injury (25, 26). However, in the second half of the 20th century, numerous deep hyperbaric treatments between 6 and 8 atm abs were performed in different centers in Hawaii (27), Hong Kong (28) or Shanghai (29). However, it is difficult to assess the efficacy of the tables, as most studies only report percentages of improvement ranging from 51% to 94%, without necessarily specifying the clinical condition at admission or at discharge from the hyperbaric center. Comparison of procedures is made even more difficult by the fact that the initial severity and treatment modalities vary enormously between hyperbaric centers (30). In France, two schools of thought have prevailed over the last few decades: the proponents of 4 atm abs Nitrox or Heliox tables for military and professional diving, and the followers of 2.8 atm oxygen tables, comparable to the US Navy T5 and T6 used by in hospitals. In our own hyperbaric center, we have been influenced by these two treatment modalities, with protocols evolving over time from 4 to 2.8 atm abs tables. These changes in management have provided us with a sufficient database to compare these procedures with the results described in this study.

The main result of our study is to show a better neurological recovery when using the initial tables at 2.8 atm abs compared to the tables at 4 atm, and this result persists when we include the potential influence of initial clinical severity as a confounding variable (4, 17).

There are very few comparative studies, an Israeli study (31) attempted to compare the use of the US Navy T6 table with the Comex 30 Heliox table in a series of 33 neurological decompression patients. This study showed no difference in clinical recovery at the end of treatment, but observed a difference in initial clinical severity, which was higher in the group treated with the Comex 30 table.

The supposed superiority of the 4 atm abs table with the Heliox mixture was not confirmed by the Danish experimental work (32) on an animal model (anesthetized rats). This model consisted of injecting bubbles into different tissues (adipose tissue, medullary white matter, muscle and tendon) in order to follow their evolution after decompression and then recompression for therapeutic purposes. Different modalities of recompression were tested at a depth of 2.8 atm abs with inhalation of air, oxygen, Heliox 80% and 50% and at 4 atm abs with Heliox 50%. This work showed that the reduction of bubbles in the white matter of the spinal cord, which is the target tissue for spinal cord DCS, was greater with oxygen recompression at 2.8 atm abs and with 50% Heliox at 4 atm abs, with no significant difference between these two methods.

Recently, a study in an animal model of spinal cord DCS highlighted the value of performing initial tables at 2.8 atm abs at 100% O2 versus higher pressure tables (33). Pigs were subjected to an insult dive at 7 atm abs for 24 min followed by rapid decompression. 61 pigs that developed neurological DCS were randomized to one of four U.S. Navy treatment tables: T6, T6A-air (21% oxygen, 79% nitrogen), T6A-nitrox (50% oxygen, 50% nitrogen), and T6A-heliox (50% oxygen, 50% helium). The authors found no significant differences among the four treatment groups. However, although the trends were not statistically significant, the T6-treated animals had the lowest rates of functional deficits and the least amount of spinal cord injury.

Our results are in line with this experimental study, suggesting a better clinical efficacy of the initial tables at 2.8 atm abs compared to the deeper tables at 4 atm abs. The efficacy of the 2.8 atm abs tables could possibly be explained by a threshold pressure level above which the effect on bubble compression does not bring any objectifiable gain, with the addition of better denitrogenation during the 2.8 atm abs tables using only 100% oxygen.

The results of our study may also suggest that there is no clinical benefit to this increase in pressure up to 4 atm abs, with a theoretical effect of pressure on bubbles that remains small compared to the reduction in bubble diameter or volume for these recompression levels between 2.8 and 4 atm abs (21).

Another hypothesis is that a high percentage of 100% O2 may not only accelerate the washout of the remaining inert gas in the tissue, but also allow the O2 to equilibrate with the inert gas in the bubble. In a second step, the O2 in the bubble is expected to diffuse into tissues with a lower O2 pressure. Metabolic consumption of O2 could thus contribute to the disappearance or reduction of these residual oxygenated bubbles by counter-diffusion (34).

Due to the risk of neurotoxicity of oxygen, increasing the pressure above 2.8 atm abs implies the use of helium or nitrogen-based diluent mixtures. In this case, the counter-diffusion effect of O2 is less pronounced and the diluent mixtures have a negative effect, contributing to a temporary increase in the inert gas load.

Taking these assumptions into account, the time spent delivering oxygen at high partial pressure, i.e., 2.8 atm (in line with accepted neurological toxicity limits for hyperbaric treatment), should have a greater therapeutic impact in this context than time spent at lower partial pressure levels. To support this hypothesis, we compared the ratio of O2 partial pressure at 2.8 atm over the total duration of the initial tables, looking for a correlation with the level of sequelae. It turns out that the higher the ratio, the lower the level of sequelae, with the best ratio for the short O2 table at 2.8 atm. However, the beneficial effect of a high partial pressure of oxygen can be detrimental if the cumulative dose of oxygen delivered during the table is too high, increasing the risk of oxygen-related toxicity.

That’s why we also considered another point based on the correlation we found between the cumulative oxygen dose (UPTD) delivered by the different initial tables and the sequelae score, indicating lower sequelae at lower oxygen doses. Several methods of determining pulmonary oxygen toxicity have been studied. One of the best known is the Unit Pulmonary Toxic Dose (UPTD), which is based on the decrease in vital capacity after dry hyperbaric exposure in resting subjects. The threshold of 615 UPTD is usually considered a safe daily limit for dry hyperbaric exposure. The UPTD is also used to compare therapy tables in terms of cumulative O2 dose delivered (35). The Comex 30 table was found to have the highest oxygen dose (923 UPTD), compared to the long (703 UPTD) or short (377 UPTD) 2.8 atm abs tables in our study. Indeed, the Comex 30 table has recently been shown to expose patients to oxygen-related pulmonary toxicity, manifested by both transient respiratory symptoms and elevated markers of pulmonary inflammation (35).

The onset of this pulmonary inflammatory state may be one of the factors explaining the reduced efficacy of these tables. In fact, during the crucial first 24 h, the organism, initially attacked by the formation of post-decompression bubbles, reacts by activating several cascades that generate a generalized immuno-inflammatory state.

In this context, an increase in the inflammatory state associated with hyperbaric treatment must be avoided at all costs. For this reason, 2.8 atm abs tables, similar to USN T5 or T6 tables, shorter than Comex 30, seem preferable. In addition, these 2.8 abs atm oxygen tables are able to inhibit leukocyte adhesion and reperfusion injury (36), which is not documented with the Heliox 50% table at 4 abs atm.

Taking the results of the two correlations together, it appears that the benefit of tables in the treatment of DCS corresponds to a balance between the ability to deliver O2 at a high partial pressure, i.e., 2.8 atm, and a limited exposure time in order to deliver a cumulative dose of oxygen that does not lead to pulmonary inflammation associated with O2 toxicity.

#### Short vs. long 2.8 atm abs tables

Regarding the duration of recompression in a cohort of patients with comparable initial severity, 15% of patients had sequelae with a short 2.8 atm table versus 41% with a long table, with a significant difference in mJOA scores.

Short tables at 2.8 atm abs are generally not recommended for the treatment of severe neurological DCS, yet their supposedly lower efficacy remains to be demonstrated.

However, the treatment of DCS with a single short table at 2.8 atm abs has long been developed in the United States for the hyperbaric chamber of construction sites. These short tables (2.5 h) at 2.8 atm abs seem to give good results in the published series. Hart et al. (37) note 79%–95% recovery depending on the clinical form for 77 DCS treated with the Hart-Kindwall table. Cianci & Slade (38) found 97.5% recovery in a series of 140 neurological DCS with cerebral signs, including cognitive signs, recompressed in the 48 h.

Recently, a prospective randomized Australian study concluded that a short table at 2.8 atm abs was more effective than the USNT6 table (39). However, this study only involved mild DCS without neurological impairment.

Furthermore, as previously discussed, these short tables at 2.8 atm abs are likely to generate less pulmonary inflammation linked to pulmonary oxygen toxicity than longer tables (35).

From our point of view, determining the optimal duration of the initial table at 2.8 atm abs for the management of neurological DCS is an issue that needs to be studied, particularly for the most severely affected patients.

### Additional hyperbaric treatment in the first 48 h

Our study suggests that additional tables at 4 atm abs may be less effective than tables at 2.8 atm abs (Heliox 50%) or 2.5 atm abs (O2 100%).

After initial recompression, a certain number of patients do not recover, and we thought it appropriate to provide additional specific hyperbaric treatment within the first 24–48 h, based on the hypothesis that this period corresponds to the peak of post-ischemic phenomena after initial reperfusion. For example, it has been emphasized that deleterious processes such as leukocyte activation are observed in DCS (19, 20). Therefore, in addition to the physical effects on bubbles and oxygenation, HBOT sessions may also be of interest in inhibiting leukocyte adhesion or other anti-inflammatory processes (36).

Furthermore, we believe that the use of helium in combination with HBOT may also activate complementary neuroprotective effects. Therefore, over the years we have developed different protocols for additional tables with 50% Heliox at 4 atm abs and more recently at 2.8 atm. However, our study suggests that additional tables at 4 atm may be less effective than tables at 2.8 atm (Heliox 50%) or 2.5 atm (O2 100%). This lower effectiveness of 4 atm tables may also be related to the previously mentioned problem of inflammation associated with pulmonary O2 toxicity, which favours the use of secondary tables with lower partial pressures after the first table (35).

Currently, a complementary recompression protocol with a lower partial pressure of O2, including Heliox 50% tables at 2.8 atm, is used in the event of an unsatisfactory neurological outcome of DCS. The neuroprotective effects of helium have not been clearly identified and may be mediated by induced hypothermia (40), antithrombotic effects (41), inhibition of apoptosis (42) and stimulation of neoangiogenesis (43). Our study does not formally conclude that these additional Heliox sessions are more effective than 100% O2 HBOT sessions. However, subgroup analysis shows that for patients with equivalent 24-h severity criteria, 57% of patients in the group using the Heliox 2.8 atm abs tables had sequelae, whereas 100% of patients in the group using the 2.5 atm abs tables had sequelae, a result approaching statistical significance (*p* = 0.06).

The study by Drewry and Gorman (44) also suggests that the use of 2.8 atm abs Heliox 50% vs. 2.5 atm abs O2 100% HBOT tables may reduce the number of patients requiring additional HBOT sessions used as an efficacy criterion (9 subjects/25 vs. 20/31). However, these partial results, based on a small series (only abstracts have been published), should be confirmed.

It would be interesting to carry out a prospective study comparing these complementary sessions with Heliox or O2 100%, which would allow us to know if the use of helium really brings a benefit. Other gases could also be good candidates to optimise the neutralisation of neurotoxic cascades with anti-NMDA agents such as xenon or argon in combination with HBOT (42, 45–49).

### Drug therapies

Very few studies have been conducted to evaluate the efficacy of drug treatments in DCS in humans. Only one randomized controlled trial in humans suggests the value of using a non-steroidal anti-inflammatory drug, tenoxicam, based on the number of additional HBOT sessions performed as a primary endpoint (9).

In fact, drug prescription in hyperbaric centers is mainly based on pathophysiological knowledge and animal DCS model studies (11, 21). Our study did not allow us to analyse the effects of the treatments commonly used in our centre, namely vascular filling with isotonic saline and the use of low-dose intravenous corticosteroids.

However, we were able to assess the effect of lidocaine, which appeared to be associated with a higher risk of neurological sequelae in the univariate analysis, although this was not confirmed after adjustment. In an additional analysis, we found that lidocaine was more frequently used in patients with high clinical severity at 24 h (72%), with a still very high rate of sequelae (87%) despite treatment. Its cardiac and neurological toxicity, narrow therapeutic margin and lack of efficacy on neurological prognosis highlighted in our study are arguments against its continued use in severe spinal cord DCS.

Fluoxetine is a selective serotonin reuptake inhibitor that is prescribed for an extended period of 3 to 6 months. This antidepressant treatment contributes to the psychological support of sequelae patients who are secondarily transferred to rehabilitation centers for long months. We also prescribe this treatment for its neuroprotective effects reported in the DCS animal model (13, 14), with an improvement in clinical recovery in ischemic stroke reported in humans (50). This molecule is used not only for its antidepressant effect, but also for anti-inflammatory purposes via the interleukin pathway acting on the central nervous system (13). Our study found no effect of fluoxetine on neurological prognosis at discharge from the hyperbaric center. However, we do not have information on the extent of long-term neurological sequelae. To determine the true effect of fluoxetine, we would need to conduct a study evaluating neurological recovery at least 6 months after hyperbaric treatment.

### Limitations

The limits of our study lies in its retrospective and mono-centric nature as well as in the small number of patients tested due to the low prevalence of this pathology.

Different protocols have indeed been applied over this 10-year period within the hyperbaric medicine service. These different management modalities can be considered as the main limitation of this study, however it is thanks to this diversity of practice that we were able to compare the different drug treatments and the hyperbaric tables performed, taking into account the main variables that would have influenced the comparison such as the initial clinical severity.

To investigate potential biases related to the retrospective nature of the study, we performed additional statistical analyses, particularly to account for initial clinical severity, which may influence the level of sequelae and potentially the effect of hyperbaric treatment. Although the result was not statistically significantly different, the mean MEDSUBHYP scores for the 2.8 atm abs tables were lower than those for the 4 atm abs tables. This is explained by the inclusion of a certain number of patients whose initial severity on admission was lower, but who deteriorated within the first 24 h. To account for this possible source of bias, we performed an overall analysis of covariance as well as subgroup analyses of patients with high and comparable initial severity. The results of these complementary analyses confirm that the initial tables at 2.8 atm abs are associated with better clinical recovery than the tables at 4 atm abs, and the short tables at 2.8 atm are also associated with better results than the long tables at 2.8 atm. Overall, our multivariate analysis accounts for potential sources of bias related not only to initial clinical severity, but also to time to recompression and deterioration within the first 24 h.

We did not examine the separate effects of hyperbaric treatment variables such as pressure level *per se*, oxygen partial pressure level, or the specific effects of gas mixtures. As these variables are related to the therapeutic tables chosen, specific studies would be required to disentangle the effects of these different variables. Our results therefore apply only to the test procedures described in this document, and the discussion of hypothesized mechanisms must also take into account this degree of uncertainty associated with the interrelated effect of these variables.

We are aware that patients with brain damage may also present with neurological signs similar to those of spinal cord DCS, but in order to select as homogeneous a group of patients as possible, we preferred to restrict the inclusion criteria and not to retain documented brain damage.

In this study, we do not have an additional assessment several months after hospitalization to determine neurological clinical status. We did not choose the Rankin Score, which is often used to assess neurological sequelae. In addition, our decision to dichotomize the outcome may have resulted in the loss of some information from the data set. However, we felt that it was clinically preferable to maintain this separation between patients with significant improvement or recovery and those with sequelae requiring rehabilitation center care.

## Conclusion

Our study suggests that initial recompression of patients with neurological deficits related to spinal cord DCS should be performed with oxygen tables at 2.8 atm abs rather than at 4 atm (Heliox 50% Comex 30). Optimal treatment appears to be a balance between the ability to deliver O2 at a high partial pressure of 2.8 atm and a limited exposure time that does not result in the pulmonary inflammation associated with O2 toxicity. Short tables at 2.8 atm (equivalent to USNT5) appear to provide a better clinical response than long tables (equivalent to USNT6), with the limitation that the comparison could not be made for the most severe patients.

**Figure 9:**
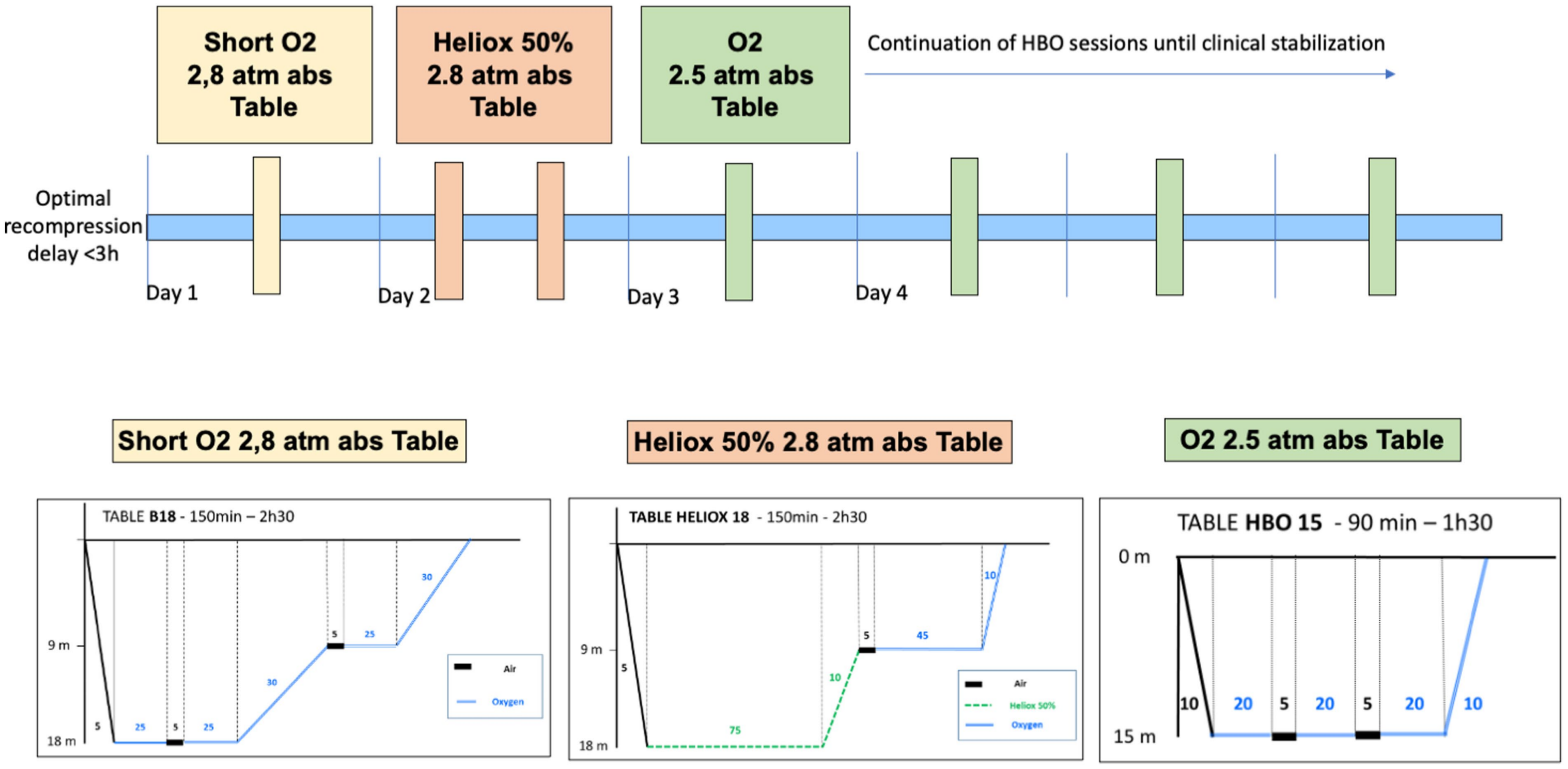
Proposed hyperbaric therapeutic management of spinal cord DCS based on results suggested by this study.

If there is no response after the first table, our study also suggests one or two additional sessions of Heliox 50% tables at 2.8 atm within the first 24–48 h (Figure 9).

Furthermore, our study shows that the risk of sequelae is not only related to the initial severity, but also to the clinical deterioration in the first 24 h, suggesting the activation of biological cascades that are not stopped by the initial recompression. We believe that the application of hyperbaric treatment must take into account this immuno-inflammatory state by trying to determine the appropriate dose, depending not only on the pressure and duration of treatment, but also on the partial pressure of oxygen and the specific potential neuroprotective effect of certain therapeutic gases, such as helium. Clearly, studies are needed to measure the immuno-inflammatory responses of patients and to understand the effects of hyperbaric therapy on these secondary processes associated with decompression sickness.

## Data availability statement

The raw data supporting the conclusions of this article will be made available by the authors, without undue reservation.

## Ethics statement

The studies involving humans were approved by Commission de validation des études cliniques HIA Sainte Anne Toulon. The studies were conducted in accordance with the local legislation and institutional requirements. The ethics committee/institutional review board waived the requirement of written informed consent for participation from the participants or the participants’ legal guardians/next of kin because study only based on retrospective data.

## Author contributions

BS and J-EB: conception and design of the study, analysis and interpretation of the data, and drafting and revising the manuscript. BS, RR, HL, JM, AD, LD, PL, SM, EG, NV, and J-EB: interpretation of the data and revising the manuscript. All authors contributed to the article and approved the submitted version.

## Conflict of interest

The authors declare that the research was conducted in the absence of any commercial or financial relationships that could be construed as a potential conflict of interest.

## Publisher’s note

All claims expressed in this article are solely those of the authors and do not necessarily represent those of their affiliated organizations, or those of the publisher, the editors and the reviewers. Any product that may be evaluated in this article, or claim that may be made by its manufacturer, is not guaranteed or endorsed by the publisher.
